# Gasoline Vapor Emissions During Vehicle Refueling Events in a Vehicle Fleet Saturated With Onboard Refueling Vapor Recovery Systems: Need for an Exposure Assessment

**DOI:** 10.3389/fpubh.2020.00018

**Published:** 2020-02-07

**Authors:** Jenni A. Shearston, Markus Hilpert

**Affiliations:** Department of Environmental Health Sciences, Mailman School of Public Health, Columbia University, New York, NY, United States

**Keywords:** gasoline, environmental exposure, vehicle refueling, volatile organic compounds, gas station

## Abstract

**Background:** Gasoline contains large proportions of harmful chemicals, which can be released during vehicle refueling. Onboard Refueling Vapor Recovery (ORVR) can reduce these emissions, but there is limited research on the system's efficacy over time in an actual vehicle fleet. The aims of this study are: (1) determine the feasibility of using an infrared camera to view vapor emissions from refueling; (2) examine the magnitude of refueling-related emissions in an ORVR-saturated fleet, to determine need for an exposure-assessment.

**Methods:** Using an infrared camera optimized for optical gas imaging of volatile organic chemicals, refueling was recorded for 16 vehicles at six gas stations. Pumps were inspected for damage, refueling shut-off valve functioning, and presence of Stage II Vapor Recovery. Vehicle make/model and age were recorded or estimated.

**Results:** Vapor emissions were observed for 14 of 16 vehicles at each station, with severity varying substantially by vehicle make/model and age. Use of an infrared camera allowed for identification of vapor sources and timing of release, and for visualizing vapor trajectories.

**Discussion:** Notably emissions occurred not only at the beginning and end of refueling but also throughout, in contrast to a prior study which did not detect increases in atmospheric hydrocarbon levels mid-refueling. Future studies are vitally needed to determine the risk to individuals during typical refueling in an ORVR saturated vehicle fleet. We recommend comprehensive exposure-assessment including real-time monitoring of emitted volatile organic compounds paired with infrared gas-imaging and measurement of internal dose and health effects of gas station customers.

## Introduction

Gasoline is a complex mixture of many chemicals, several of which are known to adversely affect human health. Of particular concern are volatile aromatic hydrocarbons, including benzene, toluene, ethylbenzene, and xylene (BTEX group), which may be released during vehicle refueling (1, 2). For example, benzene is a known human carcinogen and is associated with multiple health problems, including respiratory, nervous system, and immunological conditions (3). In addition, studies evaluating non-cancer outcomes have found decreased red blood cell counts, hemoglobin, and hematocrit levels in gas station attendants (4). While some studies have evaluated exposures to gasoline from vehicle refueling specifically (5–7), to our knowledge, few have been completed in the past decade. It is essential that such studies are repeated frequently and in varied geographic locations, as fuel composition, weather, climate, and pollution control strategies all impact individual exposures and can change over time.

In the United States (US), changes in regulations outlining gasoline vapor recovery during vehicle refueling have made this an especially pressing question. During refueling, gasoline vapor in a vehicle's tank is pushed into the atmosphere by the rising liquid gasoline level in the tank—unless a vapor recovery system is in place. From 1998 to 2006, the US Environmental Protection Agency (EPA) rolled out a requirement that nearly all newly manufactured vehicles be equipped with onboard refueling vapor recovery (ORVR) systems (8), which function by directing vaporized gasoline into a canister on the vehicle, thereby substantially reducing escape of vapors into the atmosphere. Briefly, this requirement was rolled out in stages, first for light duty vehicles (1998: 40% of new vehicles, 1999: 80%, 2000: 100%), then for light duty trucks and vans (2001: 40%, 2002: 80%, 2003: 100%), and finally for heavier light duty trucks (2004: 40%, 2005: 80%, 2006: 100%) and trucks with a >10,000 pounds gross vehicle weight rating (100% by 2006). By 2006, nearly all new gas-powered vehicles with <14,000 pound gross vehicle weight rating were required to have ORVR systems (8). In contrast, Stage II vapor recovery systems, which are used on gasoline pumps themselves, direct vaporized gasoline into gas station underground storage tanks through systems on the pumps. In 2012, the EPA determined that the US vehicle fleet was sufficiently saturated with ORVR that states could allow the removal of Stage II systems (8), thus making vapor recovery during refueling primarily dependent on ORVR systems.

Despite this change in regulations, limited information on the efficiency of ORVR systems is available, although the US EPA suggests they are 98% efficient and require minimal maintenance (8). A German study found no measurable increases in atmospheric hydrocarbon concentrations in a Sealed Housing for Emissions Determination (SHED) in which an ORVR-equipped vehicle was placed during refueling, although increases were detected at the beginning and end of refueling (9). Even though a study of presumably non-ORVR equipped vehicles in Mexico found older vehicles to have more evaporative emissions than newer ones (10), to the best of our knowledge, no assessment of the continuous functioning of ORVR systems to reduce emissions during vehicle refueling over the course of a vehicle's lifetime, within the conditions of an actual vehicle fleet, has been completed. It is possible that as vehicles age, hoses, seals, and other parts of the gas tank and ORVR system degrade, resulting in increased vapor emissions during refueling. Additionally, while some studies (6, 7) evaluated exposure to gasoline vapors during vehicle refueling in the US, finding evidence of benzene in blood and exhaled breath samples, those studies were completed before saturation of the US vehicle fleet with ORVR systems, and are thus likely over-estimates of exposures that may occur with ORVR systems. It is not currently known whether the amount of vapors today's population is exposed to would have similar, if any, effects.

Past studies assessing exposure from vehicle refueling used aluminum tubes as passive samplers (7) and sorbent tubes attached to pumps (6) to quantify exposure to gasoline vapors, positioned in the breathing zone of participants. However, such methods may not be able to detect the lower levels of exposure anticipated from a vehicle fleet with a 98% efficient ORVR system. Additionally, while these methods quantify environmental exposure to vapors during refueling, they are not easily used for source identification or to capture the dispersion and movement of vapors at the station. It is also not possible to use these devices to determine when during a refueling event vapors are more likely to be released (i.e., at the end vs. throughout), information which can help determine the cause of vapor release. Use of other technologies, such as an infrared camera optimized for visualizing compounds present in petroleum products, is needed to determine the sources of vapors during refueling (i.e., from exhaust, the vehicle tank, or the pump nozzle) and how they move through space. Such cameras are also fine-tuned to detect very small amounts of vapors, and thus may be invaluable in determining if exposure to gasoline vapors is occurring from ORVR equipped vehicles, warranting a more involved exposure-assessment.

Research on the functioning of ORVR in the actual US vehicle fleet over time, and thus an understanding of the quantity of vapors individuals may still be exposed to, is limited. Additionally, the tools traditionally used to assess exposure to vapors during vehicle refueling do not give a complete picture, as they lack the ability to determine vapor sources and movement. With this pilot study, we aim to determine the plausibility and usefulness of conducting a full exposure-assessment for exposures to gasoline vapors during vehicle refueling, in a vehicle fleet dependent on ORVR for vapor recovery. The objectives of this pilot study are to (1) determine the feasibility of qualitatively capturing fuel vapor emissions from vehicle refueling events in New York City (NYC) using a FLIR infrared camera designed specifically to detect volatile organic compounds present in petroleum products, and to (2) examine the magnitude of fuel vapor emissions over a range of different vehicle/ORVR system ages as a precursor to assessing the continuous functioning of ORVR systems over the lifetime of a vehicle in the actual US vehicle fleet.

## Materials and Methods

### Study Overview

A convenience sample of gas stations in Northern Manhattan, NYC, was selected for vapor release monitoring. At each gas station, a study member approached individuals just before they began refueling their vehicles and asked for verbal permission to record their vehicle tanks as the vehicle was refueled. This study is not human subjects research, as no information about individuals was obtained, and is thus not subject to IRB oversight.

A total of six gas stations were visited over the course of a single winter day. Three vehicle refueling events were recorded at each station, with the exception of one station where an attendant was present. For this station, only one vehicle refueling event was recorded. In total, *n* = 16 refueling events were recorded.

### Data Collection

An infrared camera optimized for optical gas imaging of volatile organic chemicals (FLIR model GF320; described below) and frequently used to detect leaks in petroleum refining operations, was used to record the fuel pump nozzle and external vehicle fuel tank filler pipe during each refueling session. In addition, researchers visually inspected gasoline pumps for hose damage, refueling shut-off valve functioning, and presence of Stage II Vapor Recovery systems. Researchers recorded the make and model of the vehicle when it was visible on the outside of the automobile, while year was estimated using photographs of the vehicle. Year was estimated by searching for images of the vehicle make and model, and comparing different years, especially the front and rear bumpers and headlight shape, to those shown in the photographs. When researchers could not definitively determine the year of the vehicle, the midpoint of the plausible year range was used. Vehicles were assigned a type based on the EPA Vehicle Classification system.

### Overview of FLIR Infrared Camera

The FLIR model GF320 infrared camera can detect 20 gases, including: 1-pentene, benzene, butane, ethane, ethanol, ethylbenzene, ethylene, heptane, hexane, isoprene, m-xylene, methane, methanol, methyl ethyl ketone, MIBK, octane, pentane, propane, propylene, and toluene (FLIR Systems Inc., 2017). The camera is tuned to detect very small spectral ranges, so that it can selectively visualize specific compounds that absorb or emit electromagnetic energy at that spectral range. A narrow bandpass filter is used to ensure that only gases with a strong signal in the specified infrared range are detected, and other components of the camera are built to emit very little energy, to reduce the signal-to-noise ratio. The manufacturer does not provide estimates of limits of detection of their camera, but we found that the GF320 can detect quite small vapor leakage rates, e.g., gas emissions from an unignited pocket lighter in outdoor atmospheric environments imaged from a distance of at least 2 m.

### Qualitative and Statistical Analysis

To determine how representative our convenience sample is of New York State and New York City vehicle fleet ORVR saturation, we used New York State's publicly available Vehicle, Snowmobile, and Boat Registrations database to calculate the proportion of registered vehicles in both the state and city that were gasoline powered and manufactured in 2006 or later (out of all gasoline powered vehicles), the year the EPA suggests that “essentially all” new gas-powered vehicles <14,000 pounds were manufactured with ORVR systems (8). We compared this to the proportion of ORVR equipped vehicles in our sample. In addition, we compared the median vehicle manufacturing age in our sample to that of registered vehicles in New York State and City.

Each infrared video was reviewed to identify the presence and magnitude of vaporized gasoline emitted during a refueling session. An overall qualitative description of each video was created, and patterns of vapor emission were identified and assigned to each session. Vapor origin (i.e., ambient vapors vs. vapors from the vehicle fuel tank) and the timing of vapor release was reviewed in all sessions. Representative video frames of “typical” emissions for each vehicle were extracted from the middle and end of each refueling session. The vapor plume was delineated using the brush feature in Microsoft Paint based on repeated observations of the videos, and not just a single frame, as it is difficult to identify the plume from a static image.

Exploratory statistical analysis was conducted in R version 3.5.1 (11). A logistic model was fit to obtain an association between estimated vehicle age and presence of vapor release during the middle of vehicle refueling, operationalized as a binary variable. Due to the small sample size no covariates were included in the model.

Figures were created with the tidyverse package in R (12), as well as with Inkscape (www.inkscape.org) and MATLAB (The MathWorks Inc., 2010).

## Results

A total of 16 refueling events at six gas stations were recorded. Our convenience sample was fairly representative of the estimated ORVR penetration proportion in New York State and City vehicles: according to EPA regulations 94% of our sample should have been equipped with ORVR, while for both New York State and City, we estimate that at least 81% of registered vehicles should have been equipped with ORVR. The median manufacturing year of our sample was 2013, the same as that for New York State and City.

Table 1 provides details about gas stations and vehicles. Of the six stations, only one had a Stage II vapor recovery system, and four had liquid gasoline leaking around the hose joints. Estimated vehicle age ranged from 1 to 32 years (manufacturing years 1987–2018), and several vehicle types (e.g., SUV, mid-size car) were represented in the sample. For 15 out of 16 vehicles, vehicle age and type combination indicated they were required to contain ORVR systems. The average refueling length was 86 s. Ambient temperature ranged from 33 to 41°F (0.5–5°C).

**Table 1:** Characteristics of gas stations and vehicle refueling events.

**Gas station ID**	**Stage II vapor recovery system**	**Hose joints**	**Vehicle ID**	**EPA vehicle size classification**	**Estimated model year**	ORVR mandate^*^	**Length of refueling (s)**
2	None	No leakage	29	Minicompact car	2014	Yes	66
			30	Midsize car	2005	Yes	88
			32	Standard sport utility vehicle	2013	Yes	88
3	None	Leakage	33	Midsize car	2006	Yes	76
			34	Mid-size car	2018	Yes	78
			35	Small sport utility vehicle	2013	Yes	84
4	None	Leakage	36	Mid-size car	2008	Yes	131
			37	Standard sport utility vehicle	2018	Yes	133
			38	Standard sport utility vehicle	2015	Yes	71
8	Vacuum assist	Leakage	41	Compact car	2005	Yes	72
			42	Midsize car	2016	Yes	122
			43	Midsize car	2008	Yes	66
9	None	Leakage	44	Standard sport utility vehicle	2004	Yes	56
			45	Large car	1987	No	110
			46	Midsize car	2015	Yes	106
7	None	No leakage	47	Minivan	2013	Yes	32

* *Indicates whether 100% of new vehicles were required to have included ORVR systems for the specific manufacturing year and vehicle type (i.e., light duty vehicle, light duty truck, and van, heavier light duty trucks, etc.)*.

The infrared camera was able to detect gasoline vapors during vehicle refueling. In addition, evaluation of the video files allowed researchers to identify vapor sources, pinpoint the time of vapor release during each video, and to see how the vapors moved after being emitted.

**Figure 1:**
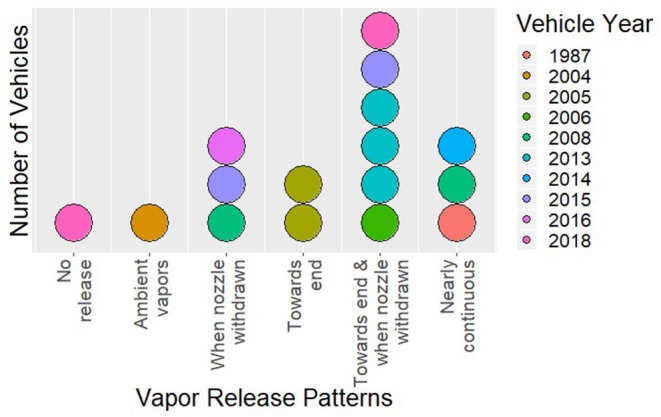
Dotplot depicting the number of vehicles in each vapor release category (each dot represents one vehicle), with year delineated by color.

Fuel vapor emissions were observed for 14 out of 16 vehicles and at every gas station. The single vehicle older than ORVR manufacturing mandates in the US clearly had much larger refueling vapor emissions than the newer vehicles. However, the majority of newer vehicles also had substantial fuel vapor emissions, particularly at the end of refueling. Qualitative descriptions of each refueling event are provided in Table 2. Six overall patterns of vapor emission were identified: no vapor release (one vehicle), ambient vapors only (one vehicle), release toward the end of refueling (two vehicles), release when nozzle was withdrawn (three vehicles), release toward the end of refueling and after nozzle was withdrawn (six vehicles), and near continuous vapor release (three vehicles). Figure 1 shows the number of vehicles in each category, and the years of the vehicles' manufacture. The three vehicles with near continuous vapor release were estimated to be 5, 11, and 32 years old. Of note, all vehicles that emitted vapors at any point during the refueling session also did so at the end of the refueling session.

**Table 2:** Qualitative description and overall patterns of vehicle refueling events.

**Vehicle ID**	**Qualitative description**	**Overall pattern**
29	Some gasoline vapor can be seen escaping into the atmosphere from the beginning of the refueling event, continuing throughout the duration of refueling. At around 0:00:41, a larger amount of vapor is seen escaping from the vehicle tank, generally increasing in amount until the end of the refueling session	Near continuous vapor release
30	No vapors are seen escaping into the atmosphere until more than a minute of refueling has passed (0:01:13), after which a large amount of vapor escapes as the vehicle tank presumably reaches full	Release toward end of refueling
32	Minimal vapor was released into the atmosphere throughout the duration of the refueling event. At the very end of refueling, as the pump is removed from the tank, a small amount of vapor can be seen escaping	Release toward end of refueling and after nozzle withdrawn
33	No vapors are seen escaping from the vehicle tank until the end of refueling, around 0:01:13, after which a large amount of vapor escapes, presumably as the tank reaches full. After the pump is withdrawn from the tank, fuel vapor continues to escape into the atmosphere in substantial quantities	Release toward end of refueling and after nozzle withdrawn
34	No vapor is seen escaping until the end of the refueling session, around 0:01:11, after which a substantial amount of fuel escapes into the atmosphere, continuing to escape even after the pump is withdrawn from the vehicle	Release toward end of refueling and after nozzle withdrawn
35	No vapor is seen escaping from the vehicle tank until the end of refueling. Vapors escape when the pump handle is partially withdrawn (0:01:12) and the tank is presumably topped off, and continue to escape even after the pump is fully withdrawn	Release toward end of refueling and after nozzle withdrawn
36	Although the pump is inserted into the vehicle from the beginning of the video, it appears that fuel is not dispensed until around 0:00:43 when the individual's hand squeezes the pump handle. As dispensing begins, large amounts of vapors can be seen escaping from the tank. Of note, the individual refueling does not fully insert the pump into the tank. Vapors escape nearly continuously throughout refueling, sometimes in large amounts. Toward the end of the session another large amount of vapor escapes, as the pump is pulled further out of the vehicle (0:01:55). Substantial amounts of vapor continue to escape until the end of refueling, including after the pump is fully withdrawn (0:02:49)	Near continuous vapor release
37	No vapor release observed	No vapor release
38	No vapor is observed until around 0:00:51, after which vapor is released nearly continuously. Vapor is observed escaping from the tank after the pump is withdrawn	Release toward end of refueling and after nozzle withdrawn
41	Some vapor is released at the beginning of the refueling session (0:00:14), but no more is observed until toward the end of refueling around (0:01:08). After this time, vapor is observed in substantial quantities until the pump is withdrawn (0:01:21), after which only minimal vapors are observed escaping	Release toward end of refueling
42	No vapors are observed until the very end of refueling, when the pump is withdrawn (0:01:59). Vapor continues to be released from the tank until it is capped	Release when nozzle withdrawn
43	No vapor release observed during refueling; a small amount of vapor may be released after pump is withdrawn (0:01:08)	Release when nozzle withdrawn
44	Poor video focus makes vapor observation difficult; however, ambient vapors appear to be present (upper right, 0:00:35, 0:00:40, 0:00:54)	Ambient vapors only
45	Substantial vapor release observed as cap is removed from tank, and continuously throughout refueling	Near continuous vapor release
46	No vapor release observed during refueling; a slight amount of release from pump observed as it was removed from tank (0:01:57)	Release when nozzle withdrawn
47	Slight amount of vapor release observed at start of refueling (0:00:03), and then again at end of refueling (0:00:24). Vapor continues to be released after pump removed	Release toward end of refueling and after nozzle withdrawn

**Figure 2:**
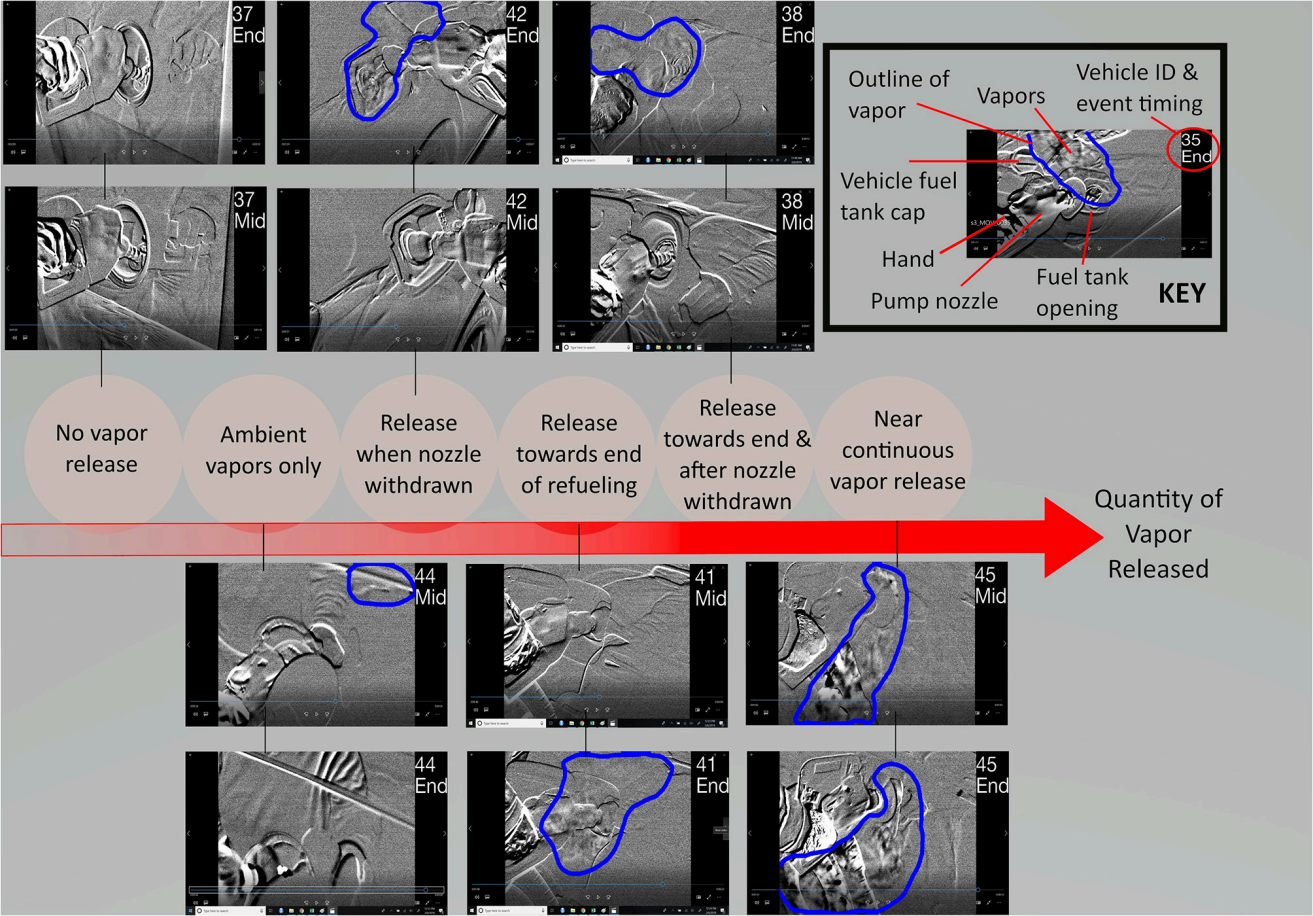
Two sample frames for each of the 6 identified patterns of vapor release during refueling: one during the middle of the refueling session, and one at the end. Vehicle ID and an indicator for middle (“Mid”) or end (“End”) of the video are included in the upper right corner of each photo. Gasoline vapors, when present, are outlined by a blue line.

Representative video frames from the middle and end of each refueling session are available in the Supplementary Material (two frames per vehicle). In Figure 2, examples from each of the six vapor emission patterns are shown, with gasoline vapor plumes delineated in blue in each frame, and vehicle IDs in the top right corner. For example, for the “release when nozzle withdrawn” category, the representative screenshot during the middle of the refueling session does not show any vapors, however, at the end of the session, vapors can be seen spilling out around the pump nozzle and the vehicle fuel tank opening. The range of emission magnitude can be seen from the various sample frames. Full video recordings for each refueling event are available at the following link: https://github.com/jenni-shearston/Vehicle_Refueling_Videos.

Results from the exploratory logistic regression were not significant, as there were not enough observations to detect an association (*n* = 16; yes release [*n* = 3]/no release [*n* = 13]). The model suggested that a 1 year increase in estimated vehicle age was associated with a 1.15 increase in likelihood of emitting vapors during the middle of refueling (95% CI = 0.97, 1.51), but this result is likely driven by the results for the 32 years old vehicle, which was much older than the rest of the vehicle population.

## Discussion

This work highlights the value of using an infrared camera to compliment more traditional methods of exposure measurement for determining potential health risks from vehicle refueling, and visually highlights the sometimes large amounts of fuel vapor emissions that occur even within an ORVR saturated vehicle fleet.

A FLIR camera allowed us to identify the source of the vapors; for example, in one video (Vehicle ID 44) vapors can be seen, but they do not originate from the pump nozzle or the vehicle tank. Of note, we observed leaking gasoline around the hose joints at this station (Station 9). For all other videos, vapors are clearly seen coming out of the pump nozzle, vehicle tank, or both. This allows for the differentiation of sources of vapor exposure, crucial information needed to intervene on exposures at gas stations generally, or to determine how effective ORVR is at minimizing vapor outflow. In addition, use of the infrared camera allowed us to confirm that vapors were emitted in a location where an individual filling up their gas tank might breathe them in (the “breathing zone”), and to visualize the dispersion and movement of the vapors. The infrared camera also made it possible to pinpoint when during a refueling session vapors were released. Sorbent tubes attached to pumps, passive samplers, and real-time monitors are not able to do this because the amount of vapor measured is averaged over a time period, so it is challenging to determine when the vapor is released, or if it is released continuously.

Information about the timing of vapor releases is particularly useful because it can help researchers determine why vapors are being released. For example, ORVR systems with “liquid seals” are known to release some vapors at the end of refueling (13), because as the flow of gasoline into the vehicle tank decreases, the air gradient into the tank created by the moving gasoline decreases, allowing vapors to flow both into the tank and out of it (and thus into the atmosphere) (9). Release at the end of vehicle refueling was indeed one of our most common observations. However, vapor releases occurring in the middle of the refueling session, or throughout the session, both of which we observed in multiple refueling events, may suggest a breakdown in functioning of the ORVR system. These findings appear to be inconsistent with the ones by Tumbrink who did not observe measurable emissions during refueling (9). Ren and Hao in China did find measurable emissions throughout refueling, but at low levels, with vapor concentration increasing over time and ranging from 0 to 4.5 mg/m^3^ (13). Emissions could be the result of a leak in part of the vehicle's fuel system, aging of the activation sites or oversaturation of the charcoal filter used in the ORVR, or a malfunctioning mechanical seal. It is also possible that that the pump nozzle itself is damaged, resulting in vapor release. In addition, Ren and Hao found that ambient temperature, fuel temperature, filling flow, and filling pipe diameter all have an impact on the time to liquid seal formation and on vapor emissions (13). Emissions were increased when either ambient or fuel temperature was higher (13). As our study was conducted at cold ambient temperatures (0.5–5°C), we expect that emissions during Spring, Summer, and Fall would be greater than what we observed.

Our study found an average refueling time of 86 s (1.43 min), similar to the 1.13 min found by Vainiotalo et al. (5) in Finland and less than that found by Egeghy et al. (7) in North Carolina (median of 3 min). These studies, and others, included various biomarkers and measures of exposure: internal dose (blood) (6), exhaled breath (7), and breathing zone air (5–7), all of which suggested individuals were exposed to benzene, a known human carcinogen, during refueling. As all studies were conducted before widespread adoption of ORVR and only at gas stations without Stage II vapor recovery, their results are likely not representative of the typical exposure today. Somewhat concerningly, however, our study suggests that despite extensive use of ORVR, individual exposures at similar magnitudes to those experienced before ORVR requirements were implemented may still occur—two of the three refueling events categorized as “near continuous vapor release” happened in vehicles manufactured after the rollout of ORVR. Without Stage II vapor recovery, the population is not protected from emissions arising from the so-called legacy fleet without ORVR, vehicles with deteriorating ORVR, or motorcycles and boats, both of which do not have ORVR.

Of particular importance for public health and policy is the ability of ORVR systems to (1) reduce exposure to gasoline vapors during refueling to a safe level, and (2) continue to function at a high level over the lifetime of a vehicle. This is important for two reasons. First, volatile organic compounds (VOCs) released during refueling can chemically react in the atmosphere, contributing to ozone and other secondary pollutant formation, which can harm human health directly through cardiovascular pathways (14). ORVR systems are intended to reduce this potential, by preventing VOCs from escaping into the atmosphere where they can react with other species. Second, as previously discussed, exposure to primary VOCs, such as those in gasoline can also negatively impact health directly, from exposure during vehicle refueling. However, limited work has been conducted to test the assumption that ORVR reduces exposure to a “safe” level during vehicle refueling. In fact, it is unclear what a “safe” level of exposure to gasoline vapors is, particularly as there is not a standardized formula for gasoline.

Numerous studies have been conducted (15, 16) to characterize the potential harms of gasoline with specific formulas or additives, but these reports typically compare different formulas of gasoline rather than comparing exposure to no exposure. Evidence suggests that while exposure during refueling is likely, health effects from gasoline at infrequent low-levels may be small, although individual components are carcinogenic (15, 16). Conversely, evidence from occupational studies has shown that individuals chronically exposed to lower levels of gasoline vapors, for example gas station attendants, are at higher risk for certain cancers (17, 18). Despite this evidence, we do not fully understand what risk gasoline vapors pose to the general public during typical vehicle refueling, or the cumulative impact of such exposure over an individual's lifetime, particularly in today's regulatory environment. Our findings highlight, in a visually compelling manner, that individuals can be exposed to substantial amounts of gasoline vapors during refueling, even in a vehicle fleet saturated with ORVR.

Future studies are vitally needed to determine the risk to individuals during typical refueling sessions in a vehicle fleet saturated with ORVR, especially because exposure to gasoline is ubiquitous and occurs throughout the lifetime. We recommend comprehensive exposure assessments that estimate exposure, internal dose, and health effects, as well as real-time monitoring of volatile organic compounds, potentially using a portable SHED (19) deployed at a gas station and paired with an infrared camera optimized for gas imaging. In addition, we recommend future work to develop an algorithm for estimating the amount or concentration of vapors shown in video from an infrared camera, to provide a better understanding of the concentration of vapors dispersing around a station.

This pilot study has several limitations. First, a convenience sample of stations and vehicles were used, and thus may not be representative of the true vehicle fleet in NYC. However, ORVR saturation in our sample was fairly close to an estimate for all registered vehicles in New York State and City (94 vs. 81%). It is additionally reassuring that both these estimates are above the EPA estimate of 71% for ORVR saturation in the older 2012 US fleet (8) and that the saturation in our convenience sample is above New York State's modeled estimate of 85% or greater for the older 2013 fleet (20). The median manufacturing year of our sample was consistent with that for New York State and City's registered vehicles (median = 2013). Second, the small sample size does not provide ample power for statistical tests. Third, vehicle make, model, and age were estimated by researchers and therefore there is potential for misclassification. Finally, real-time estimates of VOC concentrations were not obtained.

## Conclusions

In an ORVR saturated vehicle fleet, use of an infrared camera optimized for VOC imaging allowed for the identification of vapor sources, viewing vapor trajectory and dispersion, and identifying the timing of vapor release during refueling. In this pilot study, 14 out of 16 observed refueling events resulted in vapor emissions, with severity varying substantially by vehicle make/model and age. A full exposure-assessment incorporating infrared cameras, quantitative monitors, and biologic samples is needed to understand exposure to and health effects of fuel vapor at gas stations, in an ORVR saturated vehicle fleet.

## Data Availability Statement

All datasets generated for this study are included in the article/Supplementary Material.

## Author Contributions

MH and JS conceptualized the study and completed data collection. JS wrote the first manuscript draft and completed initial data analysis. MH supervised and reviewed all the data analysis and edited the manuscript. All authors agree to be accountable for the content of this work.

### Conflict of Interest

The authors declare that the research was conducted in the absence of any commercial or financial relationships that could be construed as a potential conflict of interest.
