# Supplementary figures and images for: Gasoline Vapor Emissions During Vehicle Refueling Events in a Vehicle Fleet Saturated With Onboard Refueling Vapor Recovery Systems: Need for an Exposure Assessment
Source: Front Public Health. 2020 Feb 7;8:18. doi: 10.3389/fpubh.2020.00018 (PMC7020915; doi:10.3389/fpubh.2020.00018)

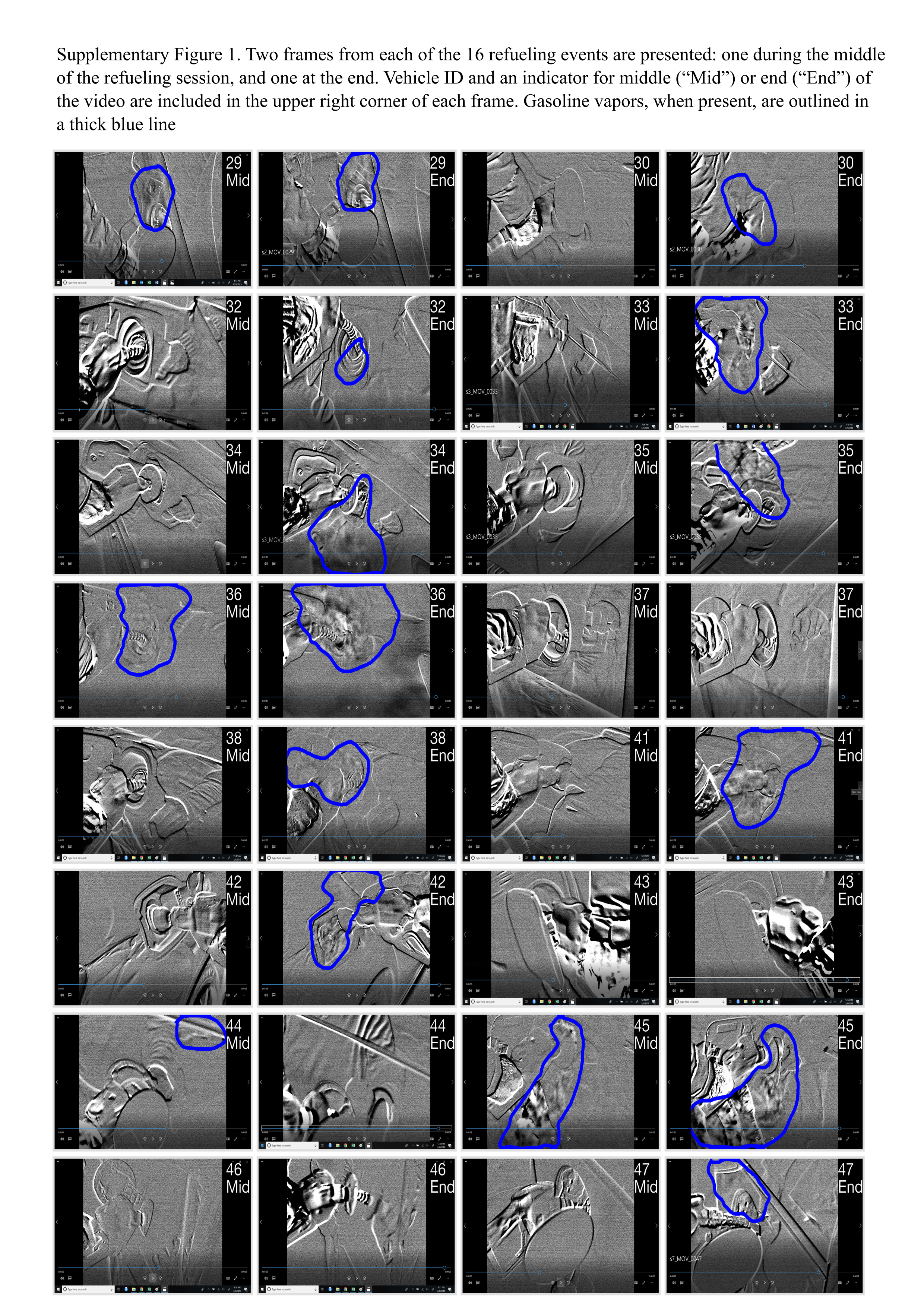

Supplement: Supplementary file 1 [file Image_1.PNG]
